# Exercise training and balance function in middle-aged and older adults with diabetic peripheral neuropathy: a GRADE-based systematic review and meta-analysis

**DOI:** 10.3389/fpubh.2026.1756867

**Published:** 2026-02-23

**Authors:** Rui Ma, Shuang Xu, Jianxin Kang, Xin Liu, Mingming Liu

**Affiliations:** 1 Hubei Sports Vocational College, Wuhan, Hubei, China; 2 Winter Olympic Academy, University of Harbin Sport, Harbin, China

**Keywords:** balance, diabetic peripheral neuropathy, disability burden, exercise, functional performance, healthy aging, meta-analysis, mobility independence

## Abstract

**Objective:**

To systematically evaluate, using the GRADE framework, the effects of exercise training on static and dynamic balance function in middle-aged and older adults with DPN.

**Methods:**

We systematically searched the Cochrane Library, EMBASE, PubMed, Web of Science, CNKI, and Scopus from inception to March 21, 2025, for randomized controlled trials (RCTs) investigating exercise interventions targeting balance in adults with DPN. Eligible studies enrolled middle-aged and older adults with DPN and reported at least one validated balance-related outcome. Risk of bias was assessed, and the certainty of evidence was rated using the GRADE approach. Meta-analyses were performed using R software and expressed as mean differences (MDs) with 95% confidence intervals (CIs).

**Results:**

Sixteen RCTs involving 759 middle-aged and older adults with DPN were included. Low to very low certainty evidence indicated that exercise training significantly improved Berg Balance Scale scores [*MD* = 2.14, 95% CI (1.57–2.73)], Functional Reach Test distance [*MD* = 3.23, 95% CI (1.82–4.64)], and Timed Up and Go test performance [*MD* = −1.65, 95% CI (−1.98–1.32)]. Exercise also increased One-Leg Stand Test duration with eyes open [*MD* = 2.93, 95% CI (2.10–3.76)] and eyes closed [*MD* = 1.37, 95% CI (0.55–2.19)]. After exclusion of a study contributing substantial heterogeneity, the Five-Times Sit-to-Stand Test showed significant improvement [*MD* = −3.07, 95% CI (−4.87–1.28)]. No significant effect was observed for the Six-min Walk Test [*MD* = 27.36, 95% CI (−18.43–73.14)].

**Conclusion:**

Exercise training may confer beneficial effects on both static and dynamic balance function in middle-aged and older adults with DPN, although the certainty of evidence is generally low to very low. No significant effect was found on six-min walking capacity. Larger, pragmatic trials are needed to confirm effects and to guide implementation in community and outpatient services, including monitoring of fall-related and longer-term functional outcomes.

**Systematic review registration:**

https://www.crd.york.ac.uk/PROSPERO/view/CRD420261305039.

## Introduction

The global prevalence of diabetes mellitus (DM) continues to rise (1, 2), with projections estimating that the number of individuals with diabetes will reach 700 million by 2045, with a higher prevalence in urban areas (3). Diabetic peripheral neuropathy (DPN) affects approximately 6% to 51% of individuals with diabetes, with its prevalence increasing with age and disease duration (4–6). DPN is a complex and progressively debilitating condition characterized by bilateral peripheral nerve dysfunction, leading to pain and sensory loss (7, 8). It can result in severe complications such as foot ulcers and amputations, significantly deteriorating overall health (9, 10). Moreover, DPN is frequently accompanied by postural instability and substantial alterations in peripheral nerve function (9). Postural instability and balance impairments are common manifestations of DPN, primarily attributed to sensory nerve dysfunction and the loss of motor axonal activity (11). The reduction in sensory input compromises proprioception, while motor nerve dysfunction contributes to muscle weakness, further impairing limb coordination and balance (8). The maintenance of balance and gait requires intricate coordination between the nervous and musculoskeletal systems. In individuals with DPN, decreased muscle strength and restricted range of motion in the ankle joint, foot, and plantar flexors are closely associated with impairments in balance and daily functional activities (12, 13). The decline in balance function places individuals with DPN at an elevated risk of falls, increasing the likelihood of functional impairments and substantially affecting their quality of life (14). Overall, balance and gait dysfunctions in DPN underscore the need for effective management strategies that support fall prevention and functional independence in aging populations (15).

Currently, pharmacological treatment is the primary approach for alleviating DPN-related pain and has demonstrated efficacy in symptom relief. However, pharmacological treatments can cause adverse effects, including sedation, weight gain, and cognitive impairment. Long-term use may also lead to dependency and tolerance, which can reduce adherence and limit overall effectiveness (16–18). In recent years, exercise interventions have gained increasing attention as a non-pharmacological strategy for DPN management. DPN-related balance impairment is largely driven by proprioceptive deficits and distal muscle weakness. Exercise programs that combine balance/proprioceptive tasks with resistance and functional training may improve sensorimotor integration and ankle–foot control. These improvements may help reduce balance impairment and fall risk. Systematic reviews have indicated that balance training, resistance training, and gait training can effectively improve balance function by enhancing lower limb strength, promoting neuroplasticity, and improving proprioception (19, 20). Particularly in middle-aged and older adults with DPN, exercise training may reduce fall risk and enhance daily functional capacity (21, 22). In 2023, the American Diabetes Association (ADA) further emphasized that improving lifestyle factors, including physical activity and behavioral interventions, can significantly enhance health outcomes and quality of life in individuals with diabetes (23). This perspective supports a broader approach to DPN management. Non-pharmacological interventions may help slow disease progression and reduce reliance on medications. This may also lower the risk of medication-related adverse effects.

Although evidence from evidence-based medicine supports the positive effects of exercise interventions on improving balance function in individuals with DPN, several limitations persist in the existing literature. First, many meta-analyses include a limited number of studies with small sample sizes, which not only weakens statistical power but also increases the uncertainty of research findings. Moreover, there is a lack of systematic analyses specifically examining the effects of exercise interventions in middle-aged and older adults with DPN. Second, considerable heterogeneity exists among studies in terms of intervention protocols, outcome measures, and study designs, which may limit the consistency and generalizability of findings. Finally, despite the diversity of balance assessment metrics, existing meta-analyses often focus on only a few dimensions, lacking a comprehensive evaluation of balance function. These limitations indicate that current evidence on the impact of exercise interventions on balance function in individuals with DPN remains insufficient, highlighting the urgent need for larger-scale and more systematic studies to advance understanding in this field. This review therefore evaluates exercise effects and evidence certainty to help inform healthy-aging initiatives, service delivery in community/outpatient settings, and future research priorities.

## Methods

This study strictly adhered to the Preferred Reporting Items for Systematic Reviews and Meta-Analyses (PRISMA 2020) guidelines and was pre-registered in PROSPERO (CRD420261305039).

### Search strategy

We searched Cochrane Library, EMBASE, PubMed, Web of Science, CNKI, and Scopus, covering studies from their inception to March 21st, 2025. The primary search terms included: Population: “Diabetic Peripheral Neuropathy” OR “DPN” OR “Diabetes-related Neuropathy”; Intervention: “Exercise” OR “Physical Activity” OR “Balance Training” OR “Resistance Training” OR “Aerobic Training” OR “Tai Chi” OR “Whole-Body Vibration Training”; Outcome: “Balance Function” OR “Postural Control” OR “Gait” OR “Falls” OR “Berg Balance Scale” OR “Functional Reach Test” OR “Timed Up and Go Test” OR “Unipedal Stance Test”; Study Design: “Randomized Controlled Trial” OR “RCT”. Two independent reviewers evaluated the eligibility of the identified publications, with disagreements resolved by a third reviewer. The detailed search strategies for each database are presented in Supplementary Table S1. Gray literature sources (e.g., conference proceedings/abstracts, dissertations/theses, and trial registries) were not systematically searched, and we restricted inclusion to peer-reviewed full-text RCTs to ensure sufficient methodological detail and extractable outcome data for risk-of-bias assessment and meta-analysis.

### Inclusion criteria

Based on the PICOS (Participants, Interventions, Comparisons, Outcomes, Study Design) framework, the inclusion criteria were as follows:

1. Participants: Middle-aged and older adults with type 1 or type 2 diabetes mellitus and clinically diagnosed diabetic peripheral neuropathy (DPN), who were able to walk independently and had no severe cognitive impairment, vestibular or central nervous system disorders, other types of peripheral neuropathy (non-DPN), or a history of foot ulcers, lower-limb amputation, or severe musculoskeletal disorders. Where reported in the original trials, we also extracted baseline characteristics such as sex, age, duration of diabetes, presence of diabetic complications, and pharmacological treatment for diabetes and DPN.
2. Interventions: Structured exercise programs designed to improve balance and/or lower-limb function, including but not limited to balance training, resistance/strength training, gait training, and multicomponent exercise interventions.
3. Comparisons: The control group received standard diabetes care and routine management.
4. Outcomes: Balance and functional performance outcomes assessed using validated clinical tests, such as the Berg Balance Scale (BBS), Functional Reach Test (FRT), Timed Up and Go (TUG), Five-Times-Sit-to-Stand test (FTSTS), One-Leg/Unipedal Stance Test (UST), and the six- min Walk Test (6MWT).
5. Study Design: Only randomized controlled trials (RCTs) were included.

### Exclusion criteria

Studies were excluded if they met any of the following conditions:

1. The study population did not meet the definition of middle-aged and older adults.
2. Studies that combined exercise with other interventions, such as cognitive therapy, were excluded.
3. Full-text or original data were unavailable.
4. Studies were duplicate publications or had unclear data descriptions that could not be extracted.
5. Mean and standard deviation (SD) were not reported, and data conversion was not possible. Authors were contacted but did not respond.
6. Non-peer-reviewed literature, including abstracts, reviews, and conference proceedings.
7. Studies published in languages other than English or Chinese were excluded. This restriction was applied for feasibility and to ensure accurate screening, data extraction, and risk-of-bias assessment based on the language expertise of the review team.

### Data extraction

Two independent researchers conducted the literature search, screening, and data extraction, followed by cross-verification of the extracted data. Any discrepancies were resolved through collective discussion within the research team and by referring to the original studies. The extracted data included the first author, year of publication, participant characteristics (e.g., age, disease duration), intervention details (type, frequency, duration), and primary outcomes (e.g., balance function, gait parameters, muscle strength indices). We extracted detailed intervention descriptions and coded each trial into predefined exercise-modality categories (aerobic, resistance, balance-oriented, and multicomponent [≥2 components]) using an a priori rule set. Two reviewers independently coded intervention modality, with disagreements resolved by consensus. Full coding rules and worked examples are provided in Supplementary Table S2.

### Risk of bias assessment

The risk of bias was independently assessed by the first and third authors. Disagreements were resolved through discussion; if consensus could not be reached, a senior reviewer adjudicated. The Cochrane Risk of Bias 2 (RoB 2) tool was used to evaluate bias across the following domains: randomization process, deviations from intended interventions, missing outcome data, outcome measurement, and selection of the reported result (24).

### Statistical analysis

All statistical analyses and visualizations were performed using R software (version 4.2.0) with the “meta” and “metafor” packages. Heterogeneity was assessed using Cochran's Q-test, and the degree of heterogeneity was quantified by the *I*^2^ statistic, with a significance level set at α = 0.10 (25). Based on the level of heterogeneity, the following models were applied: *I*^2^ < 25%: Considered low heterogeneity; a fixed-effects model was used. *I*^2^ between 25 and 75%: Considered moderate heterogeneity; a random-effects model was applied for robustness. *I*^2^^2^ > 75%: Considered high heterogeneity; a random-effects model was also used, with further exploration of potential heterogeneity sources. The primary outcome measures were continuous variables. For studies using the same measurement tools, the weighted mean difference (WMD) with a 95% confidence interval (CI) was calculated for pooled analysis. For studies using different measurement tools, the standardized mean difference (SMD) with a 95% CI was employed to estimate the effect size. To ensure result robustness, sensitivity analyses were conducted for studies with high heterogeneity to investigate potential sources of heterogeneity. Additionally, to assess publication bias, Egger's test and funnel plot analysis were performed to identify possible small-study effects or publication bias.

### Assessment of evidence quality

The quality of evidence was evaluated using the Grading of Recommendations Assessment, Development, and Evaluation (GRADE) approach, categorizing evidence as high, moderate, low, or very low. The grading criteria considered multiple factors. The risk of bias was assessed based on the included studies; if rated as “some concerns”, the evidence was downgraded by one level, while a “high risk” rating led to a two-level downgrade. Inconsistency was evaluated using heterogeneity (*I*^2^^2^), with moderate heterogeneity (> 25%) resulting in a one-level downgrade and high heterogeneity (> 75%) leading to a two-level downgrade. Imprecision was considered when results lacked statistical significance, warranting a one-level downgrade. Lastly, publication bias was assessed using Egger's test, where a *P*-value < 0.05 indicated a significant risk, leading to a one-level downgrade. This systematic approach ensures a robust evaluation of evidence quality, strengthening the credibility of the study's conclusions.

## Results

### Search results

**Figure 1:**
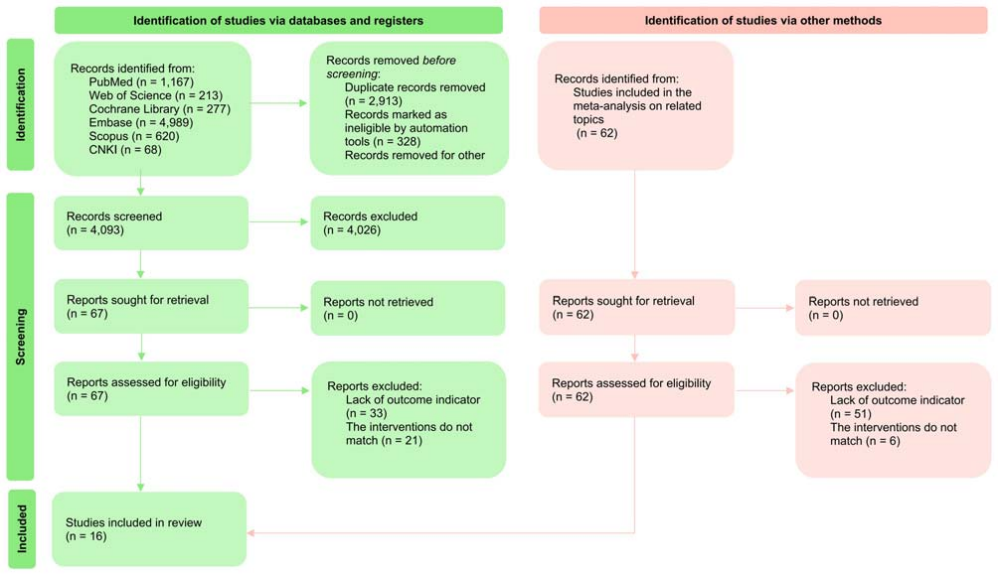
PRISMA flow diagram of study identification, screening, eligibility assessment, and inclusion.

A comprehensive search of the Cochrane Library, EMBASE, PubMed, Web of Science, and Scopus databases initially retrieved 7,266 relevant studies. After screening previously included studies from other meta-analyses, 43 potentially eligible studies were identified. Following the removal of duplicate records, 3,721 articles proceeded to title and abstract screening. Subsequently, 41 full-text articles (including studies identified from published meta-analyses) underwent detailed assessment to determine eligibility. Ultimately, 16 RCTs met the inclusion criteria and were included in the systematic review and meta-analysis. Detailed results are shown in Figure 1.

### Characteristics of included studies

A total of 16 RCTs (22, 26–40) involving 759 middle-aged and older adults with DPN were included in this study. The exercise interventions in the included studies encompassed virtual reality training (VRT), swiss ball training (SBT), resistance training (RT), balance training (BT), perceptual-motor training (PMT), aerobic training (AT), and whole-body vibration training (WBVT). The interventions primarily focused on balance, muscle strength, and gait function, employing multidimensional training approaches. The intervention duration ranged from 20 to 60 weeks, with each session lasting 20–69 min and training frequency varying from 1 to 7 sessions per week. The detailed characteristics of the included studies are presented in Table 1.

**Table 1:** Characteristics of included studies.

**Author year**	**Country**	**Age (*T*) (years)**	**Age (*C*) (years)**	**Exercise N**	**Control N**	**Frequency (sessions/ week)**	**Session duration (min)**	**Intervention duration (weeks)**	**Exercise type**	**Effect indicator**
Richardson et al. (2001) (36)	United States	64 ± 6.3	63.3 ± 7.6	9	7	7	20	3	BT	②
Lemaster et al. (2008) (33)	United States	66.6 ± 10.4	64.8 ± 9.4	38	36	3	60	48	AT,RT	⑤
Song et al. (2011) (38)	South Korea	72.9 ± 5.6	73.2 ± 5.4	19	19	2	40	8	BT	①②③④
Lee et al. (2013) (31)	South Korea	76.31 ± 4.78	75.77 ± 5.69	19	18	3	69	6	WBVT,BT	①②③④⑥
		74.05 ± 5.42		18		2	60	6	BT	
Lee et al. (2013) (32)	South Korea	73.78 ± 4.77	74.29 ± 5.2	27	28	2	50	10	VRT	①②③④⑥
Kutty et al. (2013) (29)	India	65 ± 2.12	68 ± 2.17	16	16	3	30	6	PMT	①⑤
Mueller et al. (2013) (34)	United States	65.2 ± 12.8	63.9 ± 12.5	12	10	3	60	12	BT	⑤
Quigley et al. (2014) (35)	United States	68.4 ± 9.3	67.5 ± 10.2	16	19	1	60	10	Tai Chi	①④
		67.6 ± 10.6		19		1	60	10	BT	
Yoosefinejad et al. (2015) (28)	Iran	57 ± 1.8	57 ± 1.8	10	10	2	30	6	WBVT	①
Rojhani-Shirazi et al. (2016) (37)	Iran	55 ± 5.76	53.85 ± 5.57	20	20	5	45	3	SBT	③④
		54.15 ± 4.52		20		5	45	3	BT	
Venkataraman et al. (2019) (39)	Singapore	62	62	67	67	1	20	8	BT,RT	⑥
Ahmad et al. (2019) (22)	India	66.75 ± 4.15	64.77 ± 4.6	20	17	3	55	8	PMT	①②③
Lan et al. (2019) (30)	China	67.95 ± 1.45	67.95 ± 1.45	30	30	5	30	8	AT	①
Hung et al. (2019) (27)	Taiwan	71 ± 1.22	66.5 ± 2.1	12	12	3	30	6	BT	①③④
Abdelbasset et al. (2020) (26)	Saudi Arabia	53.4 ± 5.3	52.8± 5.7	14	14	3	45	8	PMT	④⑤
Armat et al. (2024) (40)	Iran	64.27 ± 2.60	64.93 ± 2.53	25	25	4	20	4	BT	④

Exercise modalities were coded using an a priori rule set; full operational definitions and coding examples are provided in Supplementary Table S2. VRT, virtual reality training; SBT, swiss ball training; RT, resistance training; BT, balance training; PMT, perceptual-motor training; AT, aerobic training; WBVT, whole-body vibration training. Outcome measures: ① TUG; ② FRT; ③ UST; ④ BBS; ⑤ 6MWT; ⑥ FTSTS.

### Risk of bias assessment

**Figure 2:**
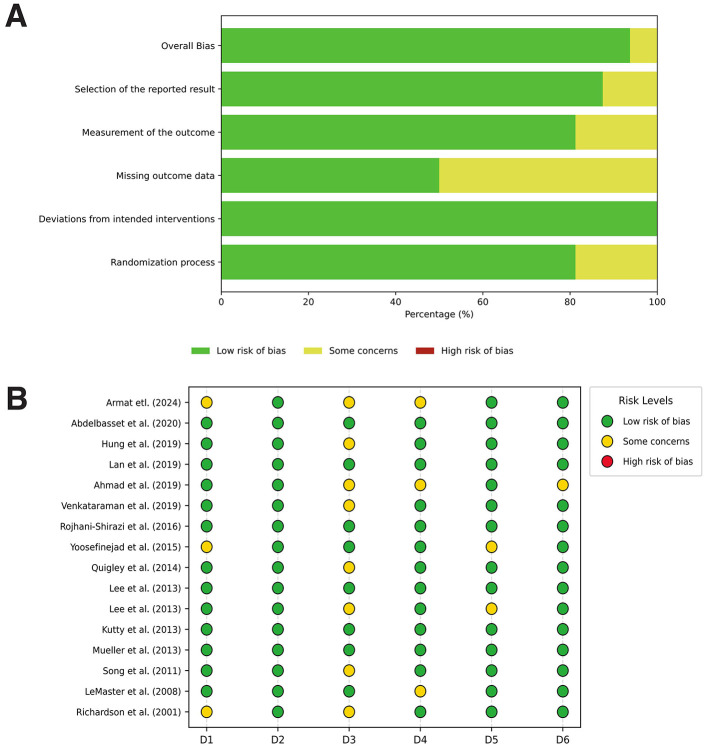
Risk of bias assessment of the included studies. **(A)** Summary plot. **(B)** Individual study assessment.

The overall risk of bias was low across most domains, with a few studies showing some concerns in the selection of reported results and missing outcome data. A small proportion of studies exhibited a high risk of bias in missing outcome data. At the study level, most included studies had a low risk of bias, with some concerns identified in reporting bias and missing data. Detailed results are presented in Figures 2A, B.

### Meta-analysis

#### 6MWT

**Figure 3:**
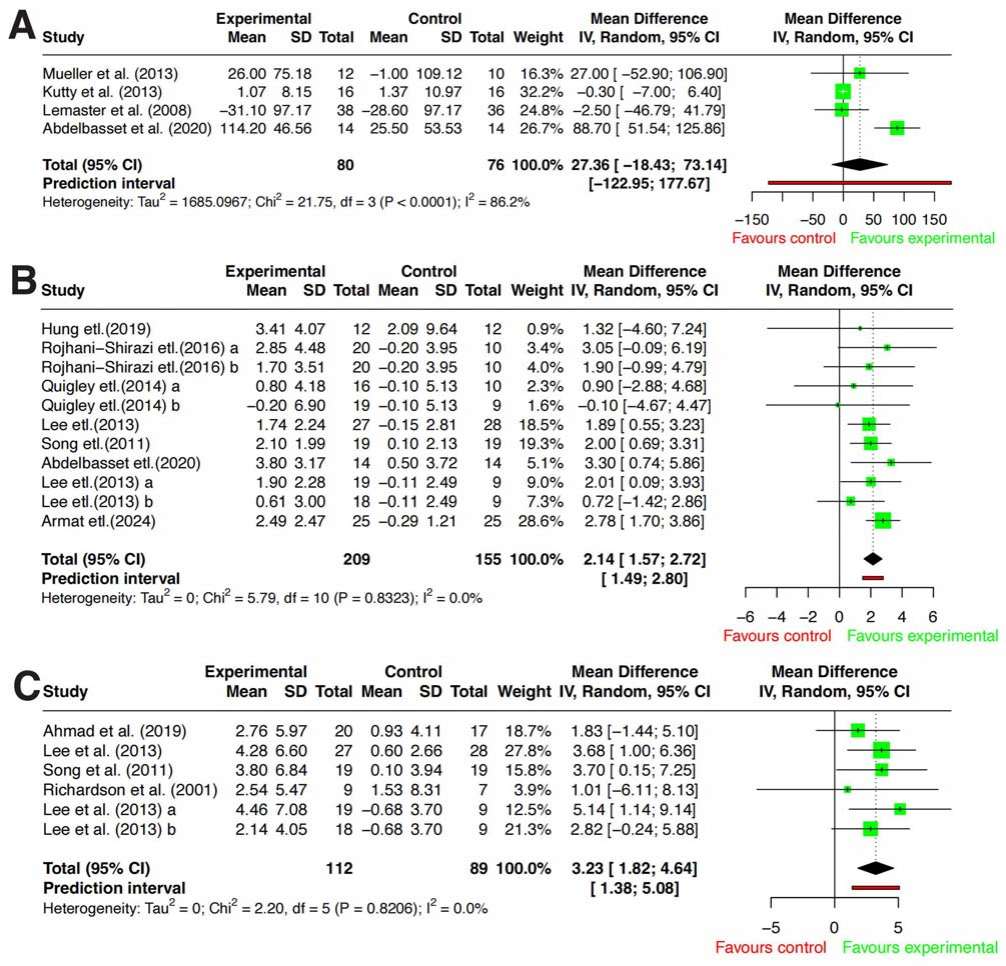
Forest plots of the effects of exercise intervention. **(A)** Six-min walk test (6MWT). **(B)** Berg balance scale (BBS). **(C)** Functional reach test (FRT). Effect estimates are presented as mean differences (MDs) calculated as exercise minus control; positive values indicate better performance in the exercise group.

A total of four RCTs (26, 29, 33, 34) were included to assess the effect of exercise interventions on 6MWT in individuals with DPN. Since all studies used the same measurement tool, WMD was used to pool the effect size. Significant heterogeneity was detected among the studies (*P* < 0.001, *I*^2^^2^ = 86.2%), prompting the use of a random-effects model for analysis. The results indicated no statistically significant difference in 6MWT scores between the exercise intervention group and the usual care group [*WMD* = 27.36, 95% CI (−18.43–73.14), *P* = 0.242]. Detailed results are presented in Figure 3A.

**Figure 4:**
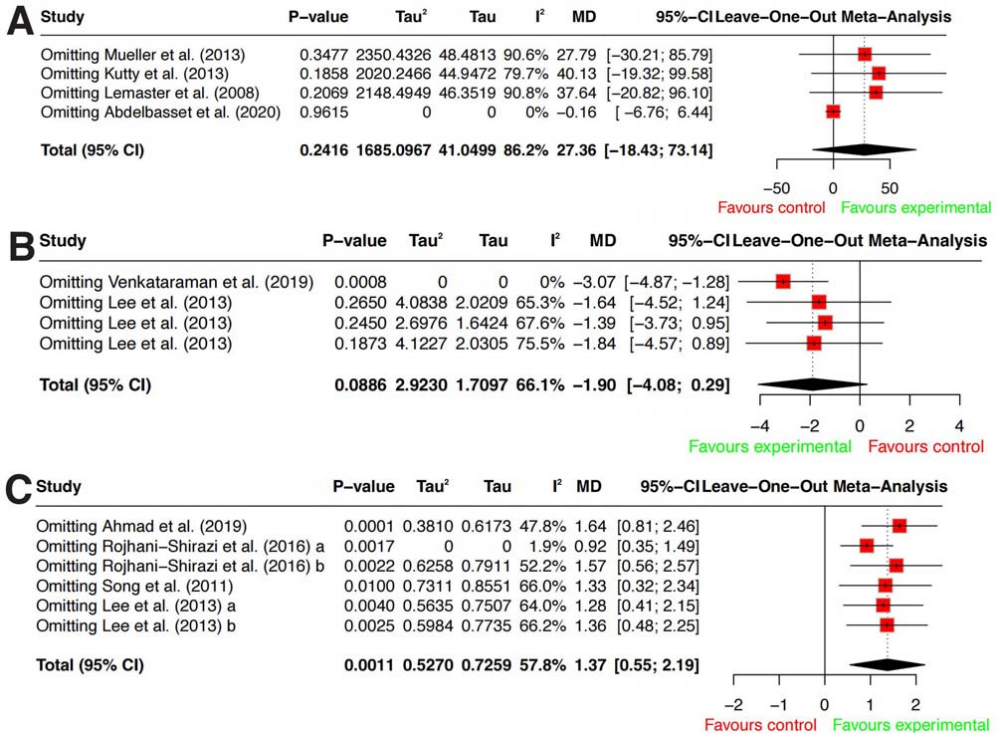
Sensitivity analysis. **(A)** Six-min walk test (6MWT). **(B)** Five times sit-to-stand test (FTSTS). **(C)** Unipedal stance test with eyes closed (UST, eyes closed). Pooled effects are shown as mean differences (MDs) calculated as exercise minus control. For 6MWT and UST (eyes closed), positive values indicate better performance in the exercise group (higher values). For FTSTS, negative values indicate better performance in the exercise group (shorter time).

Sensitivity analysis identified Abdelbasset et al. (26) as the primary source of heterogeneity. After excluding this study, *I*^2^ decreased to 0%, suggesting that its unique study characteristics or methodological design significantly deviated from the findings of other studies. Moreover, regardless of whether this study was excluded, the 95% CI of the overall effect size crossed zero, further confirming that exercise intervention did not yield a statistically significant improvement in 6MWT performance. Detailed results are presented in Figure 4A.

#### BBS

A total of eight studies (26, 27, 31, 32, 35, 37, 38, 40) were included to evaluate the effect of exercise interventions on BBS scores in individuals with DPN. Since all studies used the same measurement tool, WMD was used to pool the effect size. Heterogeneity analysis indicated extremely low heterogeneity among studies (*P* = 0.8323, *I*^2^ = 0.0%), justifying the use of a fixed-effects model for analysis. The meta-analysis results demonstrated that, compared to usual care, exercise interventions significantly improved BBS scores, with a statistically significant difference [*WMD* = 2.14, 95% CI (1.57–2.72), *P* < 0.001]. Detailed results are presented in Figure 3B.

#### FRT

A total of five studies (22, 31, 32, 36, 38) were included to evaluate the effect of exercise interventions on FRT performance in individuals with DPN. Since all studies used the same measurement tool, WMD was used to pool the effect size. Heterogeneity analysis indicated extremely low heterogeneity among studies (*P* = 0.8206, *I*^2^ = 0.0%), justifying the use of a fixed-effects model for analysis. The meta-analysis results demonstrated that, compared to usual care, exercise interventions significantly improved FRT scores, with a statistically significant difference [*WMD* = 3.23, 95% CI (1.82–4.64), *P* < 0.001]. Detailed results are presented in Figure 3C.

#### FTSTS

**Figure 5:**
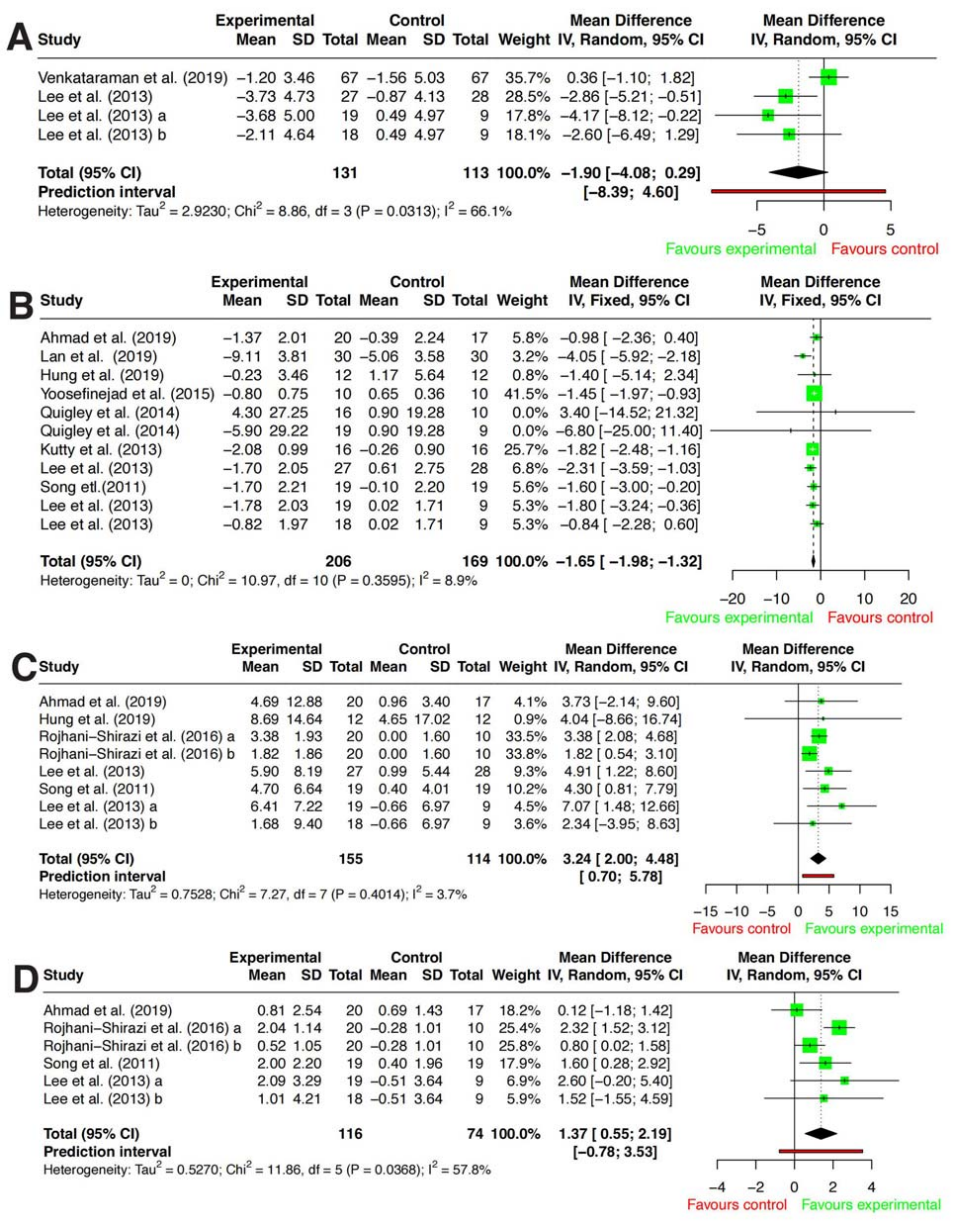
Forest plots of the effects of exercise intervention. **(A)** Five times sit-to-stand test (FTSTS). **(B)** Timed up and go test (TUG). **(C)** Unipedal stance test with eyes open (UST, eyes open). **(D)** Unipedal stance Test with eyes closed (UST, eyes closed). Effect estimates are presented as mean differences (MDs) calculated as exercise minus control. For FTSTS and TUG, negative values indicate better performance in the exercise group (shorter time). For UST outcomes, positive values indicate better performance in the exercise group (longer stance time).

A total of three studies (31, 32, 39) were included to assess the effect of exercise interventions on FTSTS performance in individuals with DPN. Since all studies used the same measurement tool, WMD was used to pool the effect size. Heterogeneity analysis indicated moderate heterogeneity among studies (*P* = 0.0313, *I*^2^ = 66.1%), prompting the use of a random-effects model for analysis. The meta-analysis results showed no statistically significant difference in FTSTS performance between the exercise intervention group and the usual care group [*WMD* = −1.9, 95% CI (−4.08–0.29), *P* = 0.886] (see Figure 4C). Detailed results are presented in Figure 5A.

Sensitivity analysis identified Venkataraman et al. (39) as a major source of heterogeneity. After excluding this study, *I*^2^ decreased to 0%, suggesting that its study characteristics or methodological design may have deviated significantly from the other studies. Following exclusion, the revised analysis indicated that exercise interventions significantly improved FTSTS performance, with a statistically significant difference compared to usual care [*WMD* = −3.07, 95% CI (−4.87–1.28)]. Detailed results are presented in Figure 4B.

#### TUG

A total of nine studies (22, 27–32, 35, 38) were included to evaluate the effect of exercise interventions on TUG performance in individuals with DPN. Since all studies used the same measurement tool, WMD was used to pool the effect size. Heterogeneity analysis indicated low heterogeneity among the studies (*P* = 0.3595, *I*^2^ = 8.9%), justifying the use of a fixed-effects model for analysis. The meta-analysis results demonstrated that, compared to usual care, exercise interventions significantly improved TUG performance, with a statistically significant difference [*WMD* = −1.65, 95% CI (−1.98–1.32), *P* < 0.001]. Detailed results are presented in Figure 5B.

#### UST (eyes open)

A total of six studies (22, 27, 31, 32, 37, 38) were included to evaluate the effect of exercise interventions on UST (Eyes Open) performance under eyes-open conditions in individuals with DPN. Since all studies used the same measurement tool, WMD was used to pool the effect size. Heterogeneity analysis indicated low heterogeneity among the studies (*P* = 0.4014, *I*^2^ = 3.7%), justifying the use of a fixed-effects model for analysis. The meta-analysis results demonstrated that, compared to usual care, exercise interventions significantly increased single-leg standing time under eyes-open conditions, with a statistically significant difference [*WMD* = 2.93, 95% CI (2.10–3.76), *P* < 0.001]. Detailed results are presented in Figure 5C.

#### UST (eyes closed)

A total of four studies (22, 31, 37, 38) were included to evaluate the effect of exercise interventions on UST (Eyes Closed) performance under eyes-closed conditions in individuals with DPN. Since all studies used the same measurement tool, WMD was used to pool the effect size. Heterogeneity analysis indicated moderate heterogeneity among the studies (*P* = 0.0368, *I*^2^ = 57.8%), prompting the use of a random-effects model for analysis. The meta-analysis results demonstrated that, compared to usual care, exercise interventions significantly increased single-leg standing time under eyes-closed conditions, with a statistically significant difference [*WMD* = 1.37, 95% CI (0.55–2.19), *P* < 0.001]. Detailed results are presented in Figure 5D.

Sensitivity analysis identified Rojhani-Shirazi et al. (37) as the primary source of heterogeneity. After excluding this study, *I*^2^^2^ decreased to 1.9%, which was substantially lower than the initial heterogeneity level (*I*^2^ = 57.8%), suggesting that its study characteristics or methodological design may have significantly deviated from the other studies. Detailed results are presented in Figure 4C.

### Publication bias

**Figure 6:**
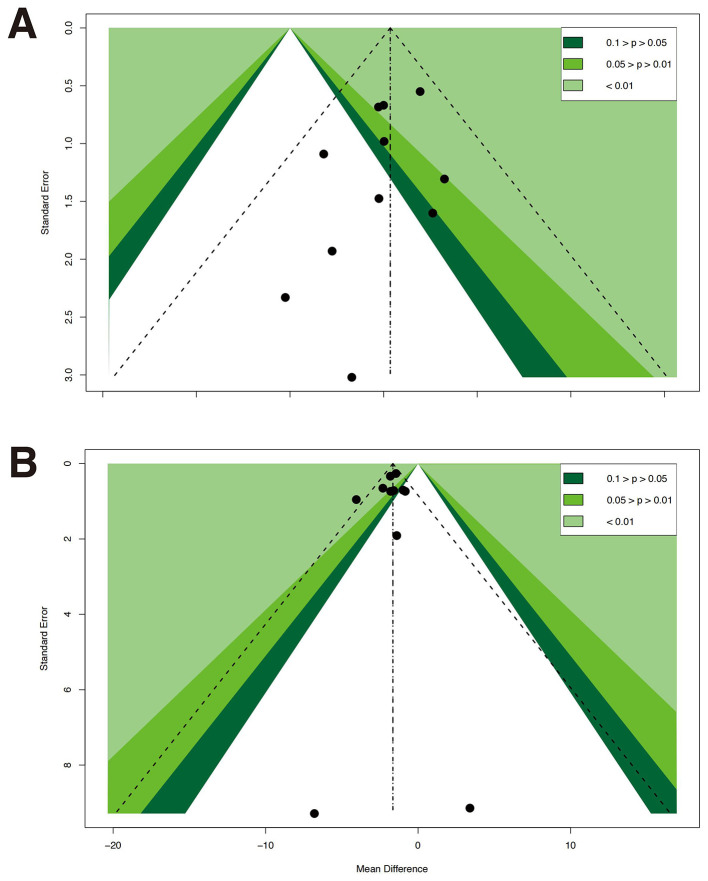
Funnel plots for publication bias assessment. **(A)** Berg balance scale (BBS). **(B)** Timed up and go test (TUG).

6MWT: Egger's test results showed *t* = 1.07, *df* = 2, *P* = 0.398, with a bias estimate of 1.9313 (*SE* = 1.8103); BBS: The funnel plot indicated that the studies were mostly symmetrically distributed, with a few data points deviating from the central axis. Detailed results are presented in Figure 6A. Egger's test results showed *t* = −1.32, *df* = 9, *P* = 0.220, with a bias estimate of −0.646 (*SE* = 0.490); FRT: Egger's test results were *t* = −0.43, *df* = 4, *P* = 0.689, with a bias estimate of −0.505 (*SE* = 1.173); FTSTS: Egger's test results showed *t* = −0.55, *df* = 9, *P* = 0.595, with a bias estimate of−0.301 (*SE* = 0.546); TUG: The funnel plot indicated a generally symmetrical distribution, although some data points deviated from the central axis. Detailed results are presented in Figure 6B. Egger's test results showed *t* = −0.55, *df* = 9, *P* = 0.595, with a bias estimate of−0.301 (*SE* = 0.546); UST (Eyes Open): Egger's test results showed *t* = −0.55, *df* = 9, *P* = 0.595, with a bias estimate of −0.301 (*SE* = 0.546); UST (Eyes Closed): Egger's test results showed *t* = 0.05, *df* = 4, *P* = 0.9621, with a bias estimate of 0.082 (*SE* = 1.62).

Overall, Egger's test did not detect statistically significant publication bias for any primary outcome (*P* > 0.05). Funnel plot analyses suggested minor asymmetry in BBS and TUG results; however, considering the statistical tests, the overall risk of publication bias was low, indicating robust study conclusions.

### Certainty of evidence

The overall quality of evidence for the relevant outcomes was rated as low to very low. The detailed results are shown in Table 2.

**Table 2:** GRADE evidence certainty assessment.

**Outcome**	**No of participants (studies)**	**Certainty assessment**					**Standardized Mean effect (95% CI)^†^**	GRADE^*^
		**Risk of bias**	**Inconsistency**	**Indirectness**	**Imprecision**	**Other**		
6MWT	156 (4 RCTs)	Some serious	Some serious	Not serious	Serious	None	27.36 (−18.43, 73.14)	Very low
BBS	364 (8 RCTs)	Some serious	Not serious	Not serious	Not serious	None	2.14 (1.57, 2.73)	Low
FRT	201 (5 RCTs)	Some serious	Not serious	Not serious	Not serious	None	3.23 (1.82, 4.64)	Low
FTSTS	244 (3 RCTs)	Some serious	Some serious	Not serious	Serious	None	−1.9 (−4.08, 0.29)	Very low
TUG	375 (9 RCTs)	Some serious	Not serious	Not serious	Not serious	None	−1.65 (−1.98, −1.32)	Low
Eyes-open UST	269 (6 RCTs)	Some serious	Not serious	Not serious	Not serious	None	2.93 (2.1, 3.76)	Low
Eyes-closed UST	190 (4 RCTs)	Some serious	Some serious	Not serious	Serious	None	1.37 (0.55, 2.19)	Very low

VRT, Virtual Reality Training; SBT, Swiss Ball Training; RT, Resistance Training; BT, Balance Training; PMT, Perceptual-Motor Training; AT, Aerobics Training; WBVT, Whole-Body Vibration Training.

Abbreviations for outcome measures: ① TUG; ② FRT; ③ UST; ④ BBS; ⑤ 6MWT; ⑥ FTSTS.

^*^GRADE, Grading of Recommendations Assessment, Development and Evaluation.

^†^CI, confidence interval.

## Discussion

### Summary of evidence

The findings of this study indicate that exercise interventions significantly improve both static and dynamic balance function in individuals with DPN. Notably, stable improvements were observed in core balance measures, including the BBS, FRT, TUG, and UST. These results support the role of exercise interventions as a crucial non-pharmacological adjunct therapy for DPN management. FTSTS, the initial meta-analysis did not show a statistically significant improvement. However, sensitivity analysis revealed that after excluding one study with high heterogeneity, the FTSTS outcome reached statistical significance, with a notable reduction in heterogeneity. This pattern indicates that the FTSTS estimate is sensitive to the inclusion of a single trial that contributed substantial heterogeneity and, given the small number of contributing studies, should be interpreted cautiously. This finding suggests that exercise interventions may enhance lower limb strength, indirectly contributing to improvements in balance function.

Regarding gait function (6MWT), neither the initial analysis nor the sensitivity analysis demonstrated significant improvements. Several factors may explain this result: Lower limb sensory and strength impairments in DPN patients may require higher-intensity or longer-duration interventions to achieve measurable improvements (41, 42). Gait function recovery depends not only on balance and muscle strength but also on cardiopulmonary fitness and overall motor coordination, which were not specifically targeted in the included interventions (8). The 6MWT is primarily an endurance-based functional test, and specialized endurance training may be required to achieve significant improvements in this measure (43, 44). Future research should explore personalized gait training programs incorporating cardiopulmonary endurance training to enhance gait function in DPN patients.

It is important to note that the overall certainty of evidence in this meta-analysis was downgraded to very low to low based on the GRADE assessment due to limitations such as imprecision, inconsistency, and risk of bias across the included studies. Given the growing burden of DPN-related falls and mobility limitation in aging populations, these ratings are important for interpreting the population-level relevance of our findings. In particular, the 6MWT and eyes-closed UST were rated as very low certainty because the evidence base was limited (few trials) and showed substantial inconsistency and/or imprecision (e.g., high heterogeneity and wide confidence intervals). As a result, the true effects may differ materially from the pooled estimates, and the current evidence does not support strong inferences about walking endurance or downstream outcomes such as falls. Therefore, while the results suggest a positive effect of exercise interventions on balance function in DPN patients, caution is warranted when interpreting these findings, particularly for outcomes rated as low or very low certainty. These downgrades were also driven by concerns in several RoB 2 domains, particularly deviations from intended interventions and selection of the reported result. In addition, protocols/registrations were not consistently available, which limited our ability to verify prespecified outcomes in some studies. Therefore, reporting bias cannot be ruled out, and the pooled effects–especially for performance-based outcomes that are susceptible to unblinded assessment–may have been overestimated. The characteristics of the included patient populations should be considered. Across the trials, participants were middle-aged and older adults with diabetes and established DPN. Most were able to walk independently and had no recent foot ulcers, amputations, or severe musculoskeletal disorders. This profile suggests relatively preserved functional capacity and, in most cases, mild-to-moderate rather than end-stage DPN. It is plausible that the magnitude of benefit from exercise differs along the spectrum of disease severity: patients with earlier or moderate DPN may experience greater gains in balance and lower-limb function, whereas those with advanced neuropathy, long-standing diabetes, or multiple complications may require more intensive, multimodal rehabilitation and may show smaller improvements. The absence of a significant effect on gait endurance (6MWT) in our meta-analysis may partly reflect the fact that gait performance in patients with more severe disease is strongly influenced by cumulative sensory loss, muscle atrophy, cardiopulmonary deconditioning, and comorbidity burden, which are not fully addressed by the exercise protocols used in the included studies. Future studies should prioritize large-scale, well-designed, multicenter RCTs to further enhance the robustness and clinical applicability of these findings (45).

### Comparison with previous meta-analyses and population-specific insights

This meta-analysis confirms the efficacy of exercise interventions in improving balance function among patients with DPN, as demonstrated by significant enhancements in the Berg BBS, TUG, and FTSTS. These results align with Streckmann et al., reinforcing the role of exercise as a key non-pharmacological intervention for enhancing balance and lower limb strength in individuals with diabetes (19, 46). However, unlike Streckmann et al., which included both pediatric and adult populations, our analysis focused exclusively on middle-aged and older adults with DPN. This narrower demographic scope likely contributed to the reduced heterogeneity observed in our study, highlighting the importance of age-specific considerations in DPN research. For the FTSTS outcome, sensitivity analysis revealed a statistically significant overall effect after excluding a study with high heterogeneity. This excluded study employed a low-frequency intervention protocol, with supervised sessions limited to once per week, whereas the remaining studies typically utilized more intensive regimens, involving at least two sessions per week. This disparity in intervention frequency likely contributed to the diminished effect observed in the initial pooled analysis, highlighting the potential importance of session frequency in optimizing exercise benefits for DPN patients.

Despite the clear benefits of exercise on balance and lower limb strength in DPN patients, its impact on gait performance, as measured by the 6MWT, remains uncertain. Hernando-Garijo et al. reported 6MWT improvements based on a single study, but their conclusion is limited by a small sample size and a specific intervention type, compromising its robustness (47). In contrast, our meta-analysis, which incorporated multiple studies, found no significant 6MWT improvement. This discrepancy may reflect insufficient exercise intensity or the absence of gait-specific interventions in the included studies, potentially limiting improvements in walking capacity. Future research should prioritize higher-intensity or gait-focused protocols to better elucidate the potential of exercise for enhancing gait function in DPN. From an aging and public health perspective, walking capacity is central to mobility independence; this has implications for public health planning, as maintaining walking capacity is closely tied to independent living and downstream health-care utilization in older adults. Health systems may consider integrating structured exercise pathways into community rehabilitation services, with outcome monitoring to ensure effectiveness across mobility domains.

### Mechanisms underlying balance impairment in DPN patients

The onset and progression of DPN are closely associated with aging, prolonged disease duration, and medication use, all of which contribute to sensory and motor nerve dysfunction (5, 48). Impairments in sensory and motor neural function are among the primary mechanisms leading to balance deficits in DPN patients (8, 11). On one hand, DPN patients often exhibit axonal atrophy, degeneration, or even complete loss of nerve fibers. Myelin sheaths undergo segmental or diffuse shrinkage or demyelination due to axonal alterations, leading to reduced proprioception and delayed neural response times. This diminished sensory perception and delayed reaction make it particularly challenging for older DPN patients to maintain postural control and dynamic balance (49). On the other hand, DPN induces muscle atrophy and weakness, particularly affecting the lower extremities, leading to reduced lower limb strength, restricted ankle and knee joint mobility, and prolonged muscle response times (50, 51). The weakening of muscle and joint function increases postural instability, causing greater sway and unsteadiness during standing and walking, ultimately compromising balance function (8, 11).

### Potential mechanisms of exercise intervention for improving balance

Exercise interventions may significantly enhance balance function in DPN patients by strengthening lower limb muscles. In this study, the significant improvement in the FTSTS suggests that increased lower limb strength may be a key mechanism underlying balance enhancement. Improved muscle strength enables patients to better manage postural transitions, particularly when performing dynamic tasks such as standing up, walking, or turning, thereby reducing the risk of imbalance and falls (52). Strengthening the lower limb muscles not only provides sufficient postural support but also enhances joint stability, further minimizing movement-related uncertainty (50, 53).

The significant improvements in dynamic balance function (evidenced by TUG and FRT) may be closely related to neuromuscular control optimization (39). Repetitive gait training and postural stability exercises facilitate adaptive neuromuscular responses, enabling DPN patients to adjust more efficiently to external changes, reduce neural response times, and enhance movement coordination (8, 54). Such adaptive adjustments contribute to improved dynamic balance in various complex environments (55). Additionally, exercise interventions may enhance proprioceptive input from muscles and joints, allowing patients to more accurately perceive body position and postural changes, thereby improving postural control efficiency (21).

Exercise interventions may further improve postural stability and dynamic responsiveness by promoting neuromuscular remodeling and functional restoration, thereby enhancing sensory input and neural feedback (8). However, despite the significant improvements in balance function, no statistically significant enhancement was observed in the 6MWT. This may be attributed to the fact that gait function recovery depends not only on balance and lower limb strength but also on cardiopulmonary fitness, prolonged exercise endurance, and overall motor coordination (39). DPN patients may experience limitations during prolonged walking due to cardiopulmonary insufficiency or muscle fatigue, preventing significant improvements in gait endurance. Future intervention studies should consider integrating aerobic exercise and cardiopulmonary training to enhance cardiopulmonary endurance, circulatory function, and muscle fatigue resistance, ultimately achieving comprehensive gait function optimization.

### Implications for aging and public health

At the population level, balance impairment in DPN contributes to falls, activity restriction, and loss of mobility independence in older adults, which increases disability burden and health-care utilization. The consistent improvements observed in functional balance tests (e.g., TUG and FRT, alongside BBS) may therefore be relevant to fall-prevention efforts because these measures reflect stability during transfers, turning, and forward reach–situations commonly associated with falls. However, because most included trials did not report fall incidence or long-term follow-up and the certainty of evidence was low to very low, these findings should be interpreted as proximal improvements in balance-related performance rather than confirmed reductions in falls or durable functional benefits. Future pragmatic trials and community programmes should incorporate standardized fall-related outcomes, longer follow-up, and routine monitoring of both balance and endurance-related mobility to better inform healthy-aging and service-planning strategies.

### Strengths and limitations

This study represents one of the largest meta-analyses to date assessing the impact of exercise interventions on balance function in individuals with DPN. A total of 15 high-quality RCTs involving 709 participants were included. Unlike previous studies that focused on single balance measures, this study comprehensively evaluated the effects of exercise interventions across multiple dimensions, including static balance, dynamic balance, lower limb strength, and gait function. This multidimensional approach provides more robust and reliable evidence to support clinical decision-making in this field. This study offers several methodological and research design advantages: Broad coverage of exercise interventions – Unlike previous meta-analyses that examined a single type of exercise intervention, this study included aerobic training, resistance training, and balance training, enhancing the generalizability of findings. Rigorous inclusion criteria and systematic risk of bias assessment – These methodological enhancements strengthen the reliability of the results. Sensitivity analysis to address heterogeneity – By excluding highly heterogeneous studies, particularly for FTSTS, the study conclusions were refined, increasing their clinical relevance. This multidimensional evaluation strategy provides valuable insights for developing personalized intervention programs for middle-aged and older adults with DPN.

Despite these strengths, this study has several limitations. First, key clinical variables such as diabetes type, duration of diabetes, glycaemic control, comorbidity burden, and DPN severity were reported inconsistently across the included trials. As a result, we were unable to perform meta-analytic subgroup analyses stratified by disease or DPN severity, and our pooled estimates should be interpreted as average effects in ambulatory middle-aged and older adults with DPN rather than across the full spectrum of disease severity. Second, the variation in outcome assessment time points and follow-up durations was not fully accounted for, which prevented subgroup analyses based on follow-up time and may have compromised the stability of the conclusions. Third, there was potential overlap between intervention modalities: some exercise protocols combined different types of training (e.g., traditional balance training, lower-limb resistance training, and stepping exercises), making it difficult to isolate the independent effects of specific exercise modalities on balance function. Fourth, we did not systematically search gray literature sources (e.g., conference proceedings/abstracts, dissertations/theses, or trial registries). Therefore, publication bias cannot be ruled out, and the pooled effects may have been overestimated. Moreover, concerns in RoB 2 domains–particularly selection of the reported result–may have further contributed to uncertainty in the pooled estimates. Fifth, we restricted inclusion to studies published in English or Chinese, which may have introduced language bias and led to the omission of relevant evidence published in other languages. Finally, the number of studies contributing to certain outcome measures, particularly gait function (6MWT) and FTSTS, was relatively small, leading to imprecise effect size estimates. Future research should employ multicenter RCTs with larger sample sizes, standardized intervention protocols, and more detailed reporting of disease severity and other clinically relevant characteristics to strengthen the evidence base for exercise interventions in patients with DPN.

## Conclusion

This meta-analysis suggests that structured exercise interventions can improve balance-related performance in ambulatory middle-aged and older adults with DPN, with consistent benefits observed in core measures of static and dynamic balance (e.g., BBS, FRT, TUG, and UST). No significant improvement was found for walking endurance as assessed by the 6MWT, and the overall certainty of evidence was low to very low; therefore, findings should be interpreted cautiously, particularly for endurance-related outcomes. From a healthy-aging perspective, balance impairment in DPN contributes to falls, activity restriction, and loss of mobility independence, increasing disability burden and health-care utilization. Our findings support exercise as a potentially scalable non-pharmacological component of community or outpatient fall-prevention and mobility-maintenance strategies, while highlighting important evidence gaps. Future studies should prioritize pragmatic, community-based trials with standardized follow-up and reporting of fall-related and longer-term functional outcomes, and should compare the comparative effectiveness and dose of exercise modalities (e.g., balance/proprioceptive training, resistance training, aerobic/endurance-focused training, and multicomponent programs), alongside implementation outcomes such as uptake, adherence, safety, and resource requirements.

## Data Availability

The original contributions presented in the study are included in the article/Supplementary material, further inquiries can be directed to the corresponding author.
