# Supplementary material for: Exercise training and balance function in middle-aged and older adults with diabetic peripheral neuropathy: a GRADE-based systematic review and meta-analysis
Source: Front Public Health. 2026 Feb 23;14:1756867. doi: 10.3389/fpubh.2026.1756867 (PMC12969934; doi:10.3389/fpubh.2026.1756867)
Supplement: Supplementary file 1 [file Table_1.docx]

**Supplementary:**

**Table 1:Search strategy：**

| Detailed search strategy | Search Results and Date |
| --- | --- |
| **Pubmed**：("diabetes" OR "diabetic" OR "DM" OR "DPN" OR "type 1 diabetes" OR "type 2 diabetes") AND ("neuropathy" OR "neuro*" OR "polyneuropathy" OR "diabetic neuropathy") AND ("balance" OR "proprioception" OR "postural control" OR "mobility" OR "movement" OR "motor skills") AND ("exercise" OR "training" OR "movement" OR "activity" OR "rehabilitation") AND ("elderly" OR "middle-aged" OR "older adults" OR "aging") | 1167 articles, March 21, 2025 |
| **Web of science：**TS=("diabetes" OR "diabetic" OR "DM" OR "DPN" OR "type 1 diabetes" OR "type 2 diabetes") AND TS=("neuropathy" OR "neuro*" OR "polyneuropathy" OR "diabetic neuropathy") AND TS=("balance" OR "proprioception" OR "postural control" OR "mobility" OR "movement" OR "motor skills") AND TS=("exercise" OR "training" OR "movement" OR "activity" OR "rehabilitation") AND TS=("elderly" OR "middle-aged" OR "older adults" OR "aging") | 213 articles, March 21, 2025 |
| **Cochrane Library**：("diabetes" OR "diabetic" OR "DM" OR "DPN" OR "type 1 diabetes" OR "type 2 diabetes") AND ("neuropathy" OR "neuro*" OR "polyneuropathy" OR "diabetic neuropathy") AND ("balance" OR "proprioception" OR "postural control" OR "mobility" OR "movement" OR "motor skills") AND ("exercise" OR "training" OR "movement" OR "activity" OR "rehabilitation") AND ("elderly" OR "middle-aged" OR "older adults" OR "aging") | 277 articles, March 21, 2025 |
| **Embase**：('diabetes' OR 'diabetic' OR 'DM' OR 'DPN' OR 'type 1 diabetes' OR 'type 2 diabetes') AND ('neuropathy' OR 'neuro*' OR 'polyneuropathy' OR 'diabetic neuropathy') AND ('balance' OR 'proprioception' OR 'postural control' OR 'mobility' OR 'movement' OR 'motor skills') AND ('exercise' OR 'training' OR 'movement' OR 'activity' OR 'rehabilitation') AND ('elderly' OR 'middle-aged' OR 'older adults' OR 'aging') | 4989 articles, March 21, 2025 |
| **Scopus**：TITLE-ABS-KEY ('diabetes' OR 'diabetic' OR 'DM' OR 'DPN' OR 'type 1 diabetes' OR 'type 2 diabetes') AND TITLE-ABS-KEY ('neuropathy' OR 'neuro*' OR 'polyneuropathy' OR 'diabetic neuropathy') AND TITLE-ABS-KEY ('balance' OR 'proprioception' OR 'postural control' OR 'mobility' OR 'movement' OR 'motor skills') AND TITLE-ABS-KEY ('exercise' OR 'training' OR 'movement' OR 'activity' OR 'rehabilitation') AND TITLE-ABS-KEY ('elderly' OR 'middle-aged' OR 'older adults' OR 'aging') | 620 articles, March 21, 2025 |
| **CNKI:** SU = ("diabetes" OR "diabetic patients" OR "diabetic peripheral neuropathy" OR "DPN" OR "type 1 diabetes" OR "type 2 diabetes") AND SU = ("peripheral neuropathy" OR "neuropathy" OR "diabetic neuropathy" OR "diabetic polyneuropathy") AND SU = ("exercise" OR "exercise intervention" OR "exercise training" OR "physical activity" OR "rehabilitation training" OR "workout") | 68 articles, March 21, 2025 |

Supplementary Table S2. Exercise-modality coding framework and trial-level classification

| Modality category | Operational definition (how we coded) | Typical cues in trial description | How we handled common “non-traditional” modalities | Examples from included trials (Table 1 exercise-type label) |
| --- | --- | --- | --- | --- |
| **Aerobic** | Rhythmic, continuous activities primarily targeting cardiorespiratory endurance. | walking/treadmill, cycling, aerobic class; intensity by HR/RPE; continuous bouts | If the programme is clearly endurance-focused (even if some warm-up balance is mentioned), code as **Aerobic**. | **AT** |
| **Resistance** | Progressive strengthening where the primary overload is muscular (external load/bands/machines). | resistance/strength training; sets/reps; progressive load | If resistance is the dominant component and balance is minor, code as **Resistance**. | **RT** |
| **Balance-oriented** | Training primarily targeting postural control, stability, proprioception, transfers/turning, or balance confidence; may include neuromotor or technology-assisted balance tasks. | balance tasks, stance/weight shift, reach, stepping, perturbation, proprioceptive/neuromotor training | **VRT/WBVT/PMT/SBT/Tai Chi** were coded as **Balance-oriented** unless the trial explicitly included structured aerobic or resistance components meeting the definitions above. | **BT, VRT, WBVT, PMT, SBT, Tai-Chi** |
| **Multicomponent (≥2 components)** | Any programme that clearly includes **two or more** of: aerobic + resistance + balance-oriented (each meeting the operational definitions). | combined or mixed programmes; separate aerobic + strength sessions; balance + resistance within same programme | Only coded as **Multicomponent** when ≥2 components are explicit (not just warm-up/cool-down). | **AT+RT**, **BT+RT** |
